# Response to S. Leonard Syme’s Essay

**Published:** 2004-03-15

**Authors:** Robin Roberson

**Affiliations:** National Institutes of Health, Bethesda, Maryland

To the Editor:

The essay "Social Determinants of Health: The Community as an Empowered Partner" by Dr S. Leonard Syme addresses why, in spite of all the biomedical advances and clinical interventions, most clinical studies are designed simply to assess disease and not to assess disease in people, especially those at the bottom of a social structure.

At the National Institutes of Health (NIH), where I have worked as a chemist for the last 20 years, it is a time of great expectations, tremendous growth, and a newfound enthusiasm under a newly proposed NIH roadmap, which holds the promise of translating basic research discoveries from bench to bedside. However, in my African American west Baltimore neighborhood, where I have resided for more than 20 years, that same air of promise and expectation for healthier lifestyles at the hands of current biomedical research does not exist. In fact, changing the face of biomedical research is not high on the list of most people's daily priorities.

Two Saturdays ago, as I was leaving a drug store in my neighborhood, I was privy to a conversation between two elderly black women, one of whom had either just picked up or dropped off a prescription. The very brief yet insightful exchange, in my opinion, sums up the sentiment many African Americans like myself have about today's health care system and health care providers. One lady commented to the other, "You know, I have a young doctor now and he's prescribed this new medication — just experimenting on me." The other lady responded, "Yes, indeed, that's all they're doing — just experimenting." This conversation exemplifies the lack of trust that still exists among African Americans, old as well as young, with health care and health care research.

To rid our country of the health disparities that still exist, first and foremost we must regain the trust of people most affected by health care disparity. Although these ladies may not have a clue about biomedical research, they realize that in spite of their efforts to establish good health regimes, widely accepted interventions might not resolve their health problems. As suggested by Dr Syme, we will achieve this trust only when researchers realize that people are the most valued resource in biomedical research and are humble enough to admit it.

